# Laparoscopic management of early primary abdominal pregnancy: A case report and literature review

**DOI:** 10.1097/MD.0000000000047987

**Published:** 2026-03-06

**Authors:** Jing Gao, Ruyue Li, Yue Zhang

**Affiliations:** a Technical Guidance Section, Reproductive Health Technology Guidance Service Center of Health Commission of Ningxia Hui Autonomous Region, Yinchuan, Ningxia, People’s Republic of China; b Gynecology Department, General Hospital of Ningxia Medical University, Yinchuan, Ningxia, People’s Republic of China.

**Keywords:** ectopic pregnancy, laparoscopy, primary abdominal pregnancy, ultrasound

## Abstract

**Rationale:**

Primary abdominal pregnancy, though a variant of extrauterine gestation, is exceedingly rare in clinical practice and is associated with a disproportionately high maternal mortality rate, necessitating vigilant diagnostic approaches and prompt therapeutic interventions.

**Patient concerns:**

A 35-year-old woman was diagnosed with a complete spontaneous abortion, as imaging revealed no paraovarian or parauterine abnormalities. She subsequently presented to our hospital’s gynecologic emergency department with a 3-day history of continuous, dull abdominal pain accompanied by nausea.

**Diagnoses:**

A primary abdominal pregnancy is suspected.

**Interventions:**

Laparoscopic exploration was successfully performed in our hospital to remove the products of conception.

**Outcomes:**

Histologic examination confirmed the diagnosis of an abdominal pregnancy. No postoperative complications occurred, and the patient was discharged the next few days.

**Lessons:**

For primary abdominal pregnancies with a stable hemodynamic state at an early stage (<12 weeks), laparoscopy is a safe and viable therapeutic option. If an abdominal pregnancy is strongly suspected, a meticulous ultrasound examination is essential to avoid misdiagnosis or missed diagnosis. Drug therapy is employed in the management of complex operations, high-risk procedures, and postoperative adjunct treatments.

## 1. Introduction

An ectopic pregnancy (EP) constitutes 1% to 2% of all pregnancies, with 95% of these implanting in the fallopian tube. Abdominal pregnancy is a rare type of EP, with an incidence reported to be between 1:10,000 and 1:30,000 live births. It constitutes approximately 1% of all ectopic pregnancies. An abdominal EP (AEP) is characterized by the implantation of the gestational sac within the peritoneal cavity, outside both the uterus and fallopian tubes. This condition carries a high risk of maternal morbidity and mortality. Various implantation sites have been documented, including the omentum, the pelvic and abdominal peritoneum, and abdominal organs such as the spleen, intestine, liver, and even the broad ligament.^[1–3]^ The diversity of potential sites and the rarity of this condition pose significant diagnostic and therapeutic challenges. A primary abdominal pregnancy refers to one where fertilization occurs within the abdominal cavity, whereas a secondary abdominal pregnancy results from an aborted or ruptured tubal pregnancy.

There are no established guidelines for the diagnosis and management of AEP. Early diagnosis is challenging, as patients often present with nonspecific symptoms such as abdominal pain and vaginal bleeding, which can lead to a misdiagnosis of tubal EP. It is rare for an abdominal pregnancy to reach full term, resulting in a viable fetus that survives the perinatal period.^[4]^ The primary diagnostic tools are beta-human chorionic gonadotropin (β-HCG) testing and transvaginal ultrasound, with computed tomography (CT) and magnetic resonance imaging (MRI) serving as adjuncts for further evaluation.^[5]^ Management options include surgical intervention, medical therapy with intramuscular or intralesional methotrexate, intracardiac potassium chloride, or a combination of medical and surgical management.^[6]^ Patients with indications for medical conservative therapy may be treated medically. Surgery is the preferred approach for AEP, with the goal of complete removal of the gestational sac. Placental management is particularly critical in advanced abdominal pregnancies. This article will present a case of laparoscopic management of a primary EP and conduct a systematic review of the literature.

## 2. Case information

### 2.1. Patient information

A 35-year-old woman presented to a nearby health center 5 days ago. No paraovarian or parauterine abnormalities were detected on imaging, and the case was considered to be a complete spontaneous abortion. She was then referred to our hospital’s gynecologic emergency department due to a 3-day history of continuous, dull abdominal pain accompanied by nausea. Her menstrual cycles were regular, with her last menstrual period occurring 7 weeks prior to presentation. The patient had no significant medical or surgical history, and no family history of genetic or infectious diseases. Her obstetric history included 1 spontaneous vaginal delivery and 1 missed abortion managed by vacuum aspiration. She did not have an intrauterine device (IUD) in place.

### 2.2. Clinical findings

**Figure 1.**
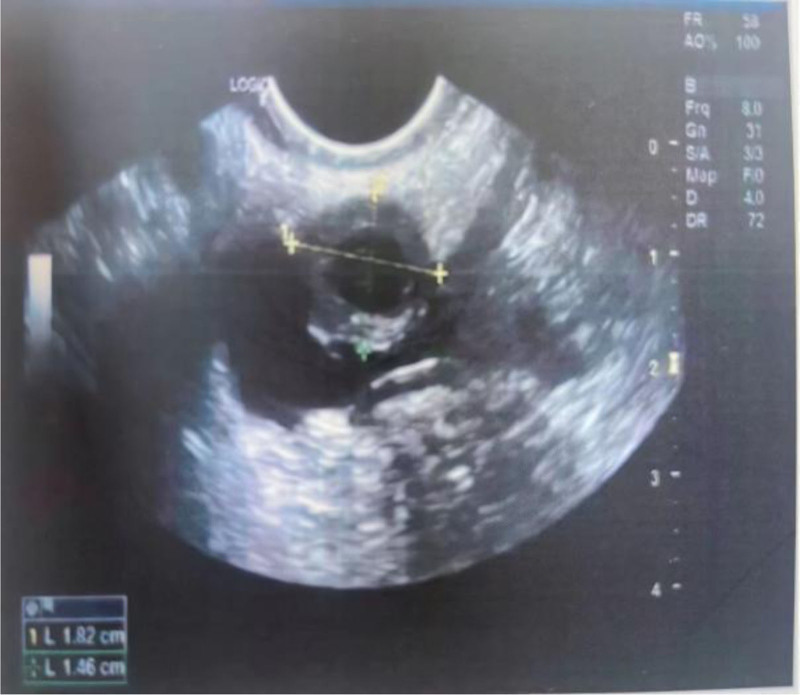
Transvaginal ultrasound scan showing a mixed-echo mass was in the middle of the pelvic cavity, measuring 18 × 22 mm.

Her vital signs were within normal limits (SpO2 98% on room air, respiratory rate 15 breaths per minute, BP 118/75 mm Hg, heart rate 74 beats per minute, and temperature 36.2°C). Physical examination revealed mild hypogastric tenderness, without rebound tenderness on deep abdominal palpation. Speculum examination and bimanual palpation showed macroscopic bleeding and elicited obvious cervical motion tenderness. Her admission hemoglobin level was 142g/L, coagulation function was normal, and the serum β-HCG was 2088 IU/L. Transvaginal ultrasonography revealed an empty uterus with an endometrial thickness of 5.5mm. A mixed-echo mass measuring 18 × 22 mm was identified in the middle of the pelvic cavity. The mass was adjacent to the peritoneum. A yolk sac was visualized within the mass, but no fetal cardiac pulsation was detected. Additionally, there was approximately 30 × 24 mm of irregular fluid in the pouch of Douglas (Fig. 1).

### 2.3. Diagnostic assessment

Due to the clinical symptoms corresponding to her sonographic findings and her β-HCG, we concluded it was an EP. Conservative management with medical treatment was not recommended due to the patient’s persistent abdominal pain. She consented to a diagnostic laparoscopy for ongoing abdominal pain and anal distension.

### 2.4. Timeline

On February 5, 2025, she was admitted to a nearby health center, where an ultrasound was performed. At 11 Am on February 10, 2025, the patient was referred to our gynecologic emergency department with continuous, dull abdominal pain, laboratory investigations and ultrasound were repeated. At 3 pm, that same day she underwent emergency exploratory laparoscopy.

### 2.5. Therapeutic intervention

**Figure 2.**
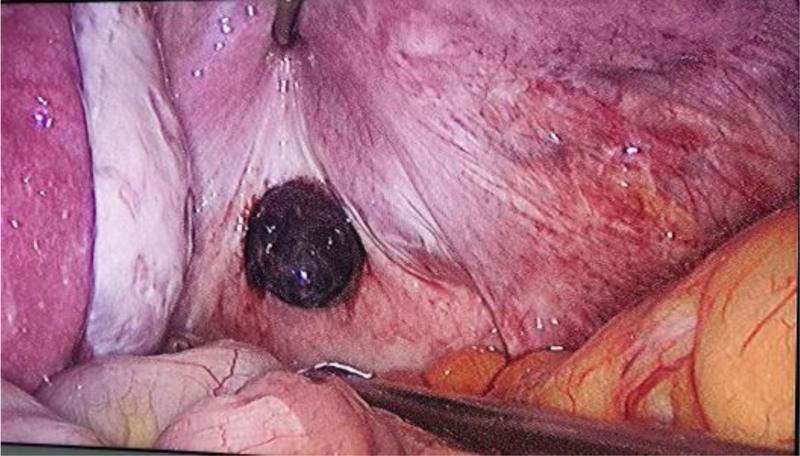
Laparoscopic view showing a mass measuring 20 × 25 mm attached to the peritoneum of the rectouterine pouch.

After entering the peritoneal cavity, we observed a small amount of free blood in the pouch of Douglas. The uterus, fallopian tubes and ovaries appeared normal in size, shape, and mobility, with no evidence of surface bleeding or tubal rupture. In the right posterior region of the uterus, attached to the peritoneum of the rectouterine pouch, a purplish-blue mass measuring 20 × 25 mm was identified (Fig. 2). This finding confirmed a primary abdominal pregnancy, ruling out a uteroplacental fistula or reimplantation following tubal abortion. The diagnosis of primary abdominal pregnancy is clear.

**Figure 3.**
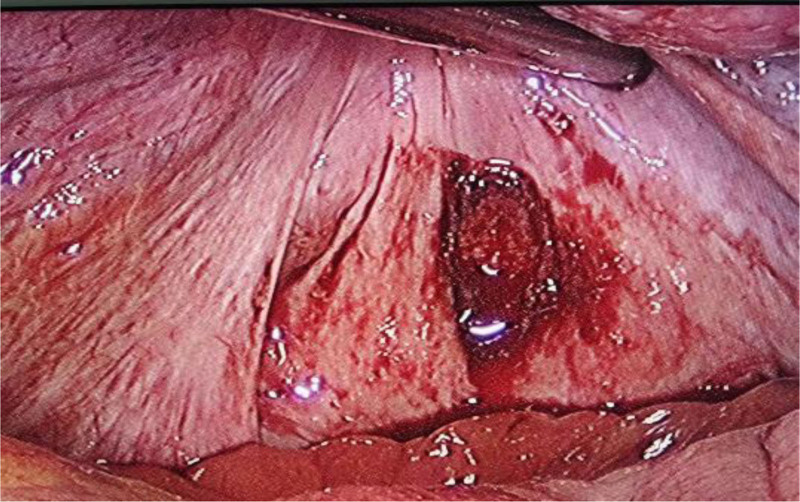
Laparoscopic view showing the gestation sac was completely removed.

**Figure 4.**
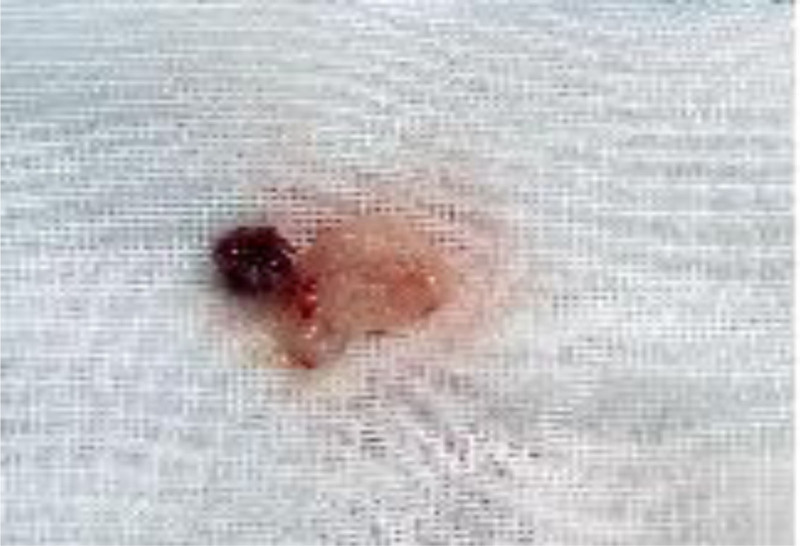
Removed pregnancy tissue after it had been rinsed with saline solution.

The gestation sac was completely removed with forceps and sent for pathological examination (Figs. 3 and 4). Moderate active bleeding occurred from the implantation site was controlled by electrocautery using bipolar electrocautery. The estimated blood loss was 10 mL.

### 2.6. Follow-up and outcomes

**Figure 5.**
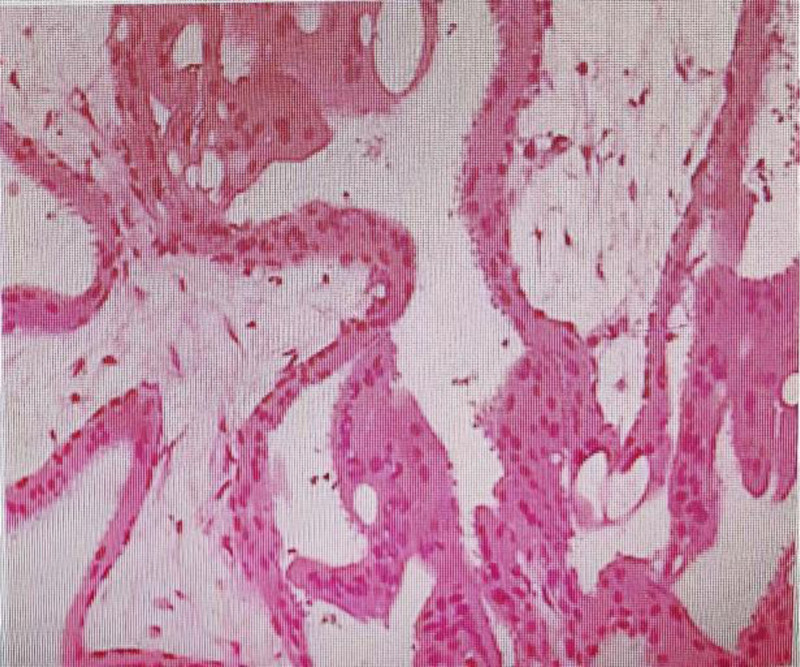
The pathological examination of the removed pregnancy tissue.

Postoperatively, a rapid decline in β-HCG levels was observed, and routine blood tests showed no abnormalities. Intraoperative pathological examination of the specimen confirmed the presence of villous tissue and blood clots (Fig. 5). The patient recovered well and was discharged in good condition on February 10, 2025. At the 1-month follow-up evaluation, both physical examination and laboratory finding s were within normal limits.

The patients and their families expressed their gratitude for the care provided throughout the treatment course. We obtained the consent of the patient and her relatives for the publication of this case.

## 3. Discussion

Abdominal pregnancy is extremely rare, accounting for 1% of all ectopic pregnancies.^[7]^ Maternal mortality is estimated to be 7.7 times higher than in other tubal pregnancies and 90 times higher than in intrauterine pregnancies.^[8]^ The potential for the gestational sac to invade internal organs and blood vessels, resulting in intra-abdominal hemorrhage, shock, disseminated intravascular coagulation, and even organ perforation and sinus formation, poses a significant risk of maternal mortality. Therefore, early diagnosis and treatment are crucial for improving patients’ clinical outcomes. In the present case, thanks to timely diagnosis and the patient’s cooperation, the procedure was performed promptly and without complications.

Based on pathogenetic mechanisms, abdominal pregnancy is classified as primary or secondary.^[9]^ Clinically, secondary abdominal pregnancy is more common. It typically results from the abortion or rupture of a tubal pregnancy, but may also be caused by uterine perforation during an abortion procedure or by a uteroperitoneal fistula, which allows the gestational sac to enter and continue developing in the abdominal cavity. The cause and mechanism of primary abdominal pregnancy remain unknown. Its diagnosis is based on Studdiford criteria,^[10]^ as follows:

1. Normal fallopian tubes and ovaries, with no signs of recent pregnancy;
2. The absence of a uteroplacental fistula;
3. Attachment of the pregnancy to a peritoneal surface, ruling out secondary implantation after a tubal abortion.^[11]^

Currently, no specific risk factors have been definitively established for abdominal pregnancy, including those commonly associated with EP such as infertility, prior EP, pelvic surgery, reproductive tract anomalies, history of sexually transmitted diseases, oral contraceptive use, or even in vitro fertilization.^[12]^ However, several studies focusing on post-in vitro fertilization abdominal pregnancy suggest potential contributing factors, which may include tubal infertility, history of tubal EP or tubal surgery, as well as the transfer of multiple or fresh embryos.^[13]^

Amenorrhea, which may occur with or without abdominal pain and vaginal bleeding, is common in early abdominal pregnancy. These symptoms often intensify as the embryo grows. AEP, unlike tubal EP, may remain undetected until an advanced gestational age. Clinical features of late abdominal pregnancy include continuous abdominal pain that worsens with fetal movement, abnormal fetal position, and a fetal presentation that is difficult to palpate despite easily palpate fetal limbs.^[14]^ Approximately 21% of newborns exhibit birth defects, including limb malformations, craniofacial anomalies, and joint abnormalities.

The patient presented with a history of amenorrhea, abdominal pain, and vaginal bleeding. Due to the atypical and nonspecific nature, these symptoms often resemble those of other ectopic pregnancies, making it challenging to confirm an abdominal pregnancy before surgery. In some instances, the condition may be misdiagnosed as a uterine pregnancy, tubal pregnancy, ruptured corpus luteum, acute appendicitis, intestinal obstruction, ovarian cyst torsion or rupture, etc.^[15]^ The diagnosis is typically established intraoperatively, in our case, it was initially diagnosed as an EP prior to surgery. Although ultrasound is the most common diagnostic method, it does not always differentiate abdominal pregnancy from other forms of extrauterine pregnancy. In fact, ultrasound permits diagnosis of early abdominal pregnancy in only 50% of cases.^[16]^ MRI can clearly display soft tissue structures, define the relationship between the gestational tissue and its surroundings, and even locate the gestational sac, thereby aiding in diagnosis and preoperative assessment. It is particularly useful for diagnosing retroperitoneal lesions that are difficult to detect by ultrasound.^[17]^ However, MRI is costly. CT is less expensive but offers inferior tissue resolution compared to MRI and also involves radiation exposure.

Surgery is the primary treatment for abdominal pregnancy, regardless of whether the diagnosis is made preoperatively. The surgical options include laparotomy and laparoscopy. Due to the high risk of uncontrolled bleeding at the implantation site, laparotomy remains the most common approach. However, in resent years, advances in technology and surgical skill have led to the increasing use of laparoscopy for diagnosing and treating ectopic pregnancies. Laparoscopy offers advantages such as shorter hospital stays, reduced blood loss, and lower invasiveness.^[15]^ Nonetheless, if an abdominal pregnancy ruptures and causes uncontrolled hemorrhage, laparotomy remains the preferred surgical method

Advanced abdominal pregnancy often remains undiagnosed until surgery, and both the mother and the fetus are at risk of fatal complications at any time. These complications frequently stem from a delayed diagnosis and inappropriate management of placenta. Placental removal should be performed with extreme caution to prevent fatal maternal hemorrhage. Angiographic arterial embolization may serve as a first-line treatment for abdominal pregnancy, aiming to avoid surgery or reduce placental vascularity, thereby making surgery safer.^[18]^ Some scholars suggest ligating the placental blood supply to facilitate its removal, if this is not feasible, the placenta may be left in situ. The patient should then be followed regularly for potential complications and to monitor for placental resorption.

Conservative treatment as the initial choice for early abdominal pregnancy is uncommon, and it has been described only in individual case reports.^[19–21]^ Medical conservative therapy may be considered under the following circumstances:

1. When the embryo is implanted at an unfavorable site associated with a high risk of severe hemorrhage or visceral injury during surgery.
2. When the diagnosis is made postoperatively following an initial surgery where the condition was missed or misdiagnosed.
3. As a supplemental treatment when serial β-HCG level show an inadequate decline after surgical intervention.^[22]^

There is no standard protocol for medical treatment. Agents such as methotrexate (administered locally or systemically), as well as local instillation of potassium chloride, hyperosmolar glucose, prostaglandins, danazol, etoposide, and mifepristone have all been reported in the literature.^[23]^ Patients require regular reevaluation and monitoring after treatment, since hemorrhage often necessitates surgical intervention.

## 4. Conclusion

Abdominal primary pregnancy is a rare form of EP, and its diagnosis is challenging due to the absence of consistent clinical features. Therefore, in cases with high clinical suspicion, a comprehensive ultrasound examinations is essential to avoid misdiagnosis. If uncertainty persists, CT or MRI may be employed. For patients who are candidates for conservative management, medical therapy is an option. Surgery remains the primary treatment. Laparoscopy is the preferred approach, as it provides superior field exposure, facilitates complete lesion removal and hemorrhage control, and can be life-saving, thereby reducing maternal and neonatal mortality. Placental management is a critical aspect of treating intermediate and advanced abdominal pregnancy. Medical therapy is typically reserved for adjunctive use postoperative, or in cases where surgery is exceptionally high-risk or complex.

## Author contributions

**Supervision:** Ruyue Li.

**Writing – original draft:** Jing Gao.

**Writing – review & editing:** Yue Zhang.
